# Elemental Influence: The Emerging Role of Zinc, Copper, and Selenium in Osteoarthritis

**DOI:** 10.3390/nu17132069

**Published:** 2025-06-21

**Authors:** Abebe Feyissa Amhare, Haobiao Liu, Lichun Qiao, Huan Deng, Jing Han

**Affiliations:** 1 Second Affiliated Hospital of Xi’an Jiaotong University, Xi’an 710004, China; abebe@xjtu.edu.cn; 2 Schools of Public Health, Health Science Center, Xi’an Jiaotong University, Xi’an 710061, China; houbiulau@gmail.com (H.L.); qlc978402409@163.com (L.Q.); denghuan0205@163.com (H.D.); 3 Schools of Nursing, Xi’an Jiaotong University Health Science Center, Xi’an 710061, China; 4 Global Health Institute, Health Science Center, Xi’an Jiaotong University, Xi’an 712000, China; 5 Key Laboratory for Disease Prevention and Control and Health Promotion of Shaanxi Province, Xi’an 710061, China; 6 Key Laboratory of Environment and Genes Related to Diseases, School of Public Health, Health Science Center, Xi’an Jiaotong University, Xi’an 710061, China

**Keywords:** osteoarthritis, trace elements, zinc, copper, selenium, cartilage, antioxidant, inflammation

## Abstract

Osteoarthritis (OA) is a prevalent and disabling joint disorder characterized by progressive cartilage degradation, subchondral bone changes, synovial inflammation, and chronic pain. While traditionally attributed to mechanical and age-related factors, increasing attention has been directed toward the role of nutritional components in disease modulation. This article critically examines the emerging role of three essential trace elements, zinc, copper, and selenium, in the pathophysiology of OA. These micronutrients are fundamental to antioxidant defense, immune modulation, and extracellular matrix (ECM) integrity. Altered systemic or local levels of zinc, copper, and selenium have been associated with oxidative stress, inflammation, and dysregulated cartilage metabolism in OA. Drawing on clinical studies, in vivo and in vitro experimental models, and population-based data, we synthesize evidence on trace element status in OA patients, mechanistic pathways, and therapeutic potential, including emerging nanomedicine strategies that enhance the targeted delivery and therapeutic efficacy of trace elements in joint tissues. This review highlights the need for integrated trace element profiling in OA research and clinical care and supports the exploration of targeted nutritional interventions in OA prevention and management.

## 1. Introduction

Osteoarthritis (OA) is a prevalent, multifactorial joint disorder that significantly contributes to pain, functional limitation, and reduced quality of life, particularly among older adults [1,2]. As the most common form of arthritis worldwide, OA affected approximately 606 million people in 2021, accounting for 7.7% of the global population [3]. The disease is primarily characterized by the progressive deterioration of articular cartilage, remodeling of subchondral bone, osteophyte (bone spur) formation, and low-grade synovial inflammation. These pathological changes collectively lead to joint stiffness, decreased range of motion, and chronic pain [2]. In advanced stages, OA may necessitate surgical intervention, including total joint replacement. The fundamental imbalance between anabolic and catabolic processes in joint tissues underlies disease progression, with chondrocytes, cartilage-resident cells, playing a pivotal role in regulating extracellular matrix turnover [4,5]. As catabolic signaling becomes dominant, matrix degradation accelerates and joint integrity deteriorates.

The etiology of OA is complex and involves mechanical loading, aging, obesity, genetic predisposition, and chronic low-grade inflammation [6]. Increasing attention has been paid to the molecular and biochemical factors underlying OA, particularly oxidative stress and inflammatory mediators, which exacerbate cartilage degradation and synovial dysfunction. These emerging insights have prompted exploration into the roles of systemic factors, including micronutrients and trace elements, in modulating disease onset and progression [7].

Essential trace elements such as zinc, copper, and selenium play crucial roles in maintaining redox homeostasis, enzymatic function, and immune regulation, which are disrupted in OA [7,8]. Zinc is a structural component of over 300 enzymes and plays a vital role in matrix metalloproteinase (MMP) activity, which is directly involved in cartilage breakdown [9,10]. Copper is essential for lysyl oxidase-mediated collagen and elastin cross-linking, and it also supports the antioxidant activity of enzymes like superoxide dismutase (SOD) [7,11]. Selenium plays a crucial role in human health through its incorporation into selenoproteins, particularly in the form of selenocysteine. There are 25 selenoproteins in humans, many of which are involved in antioxidant defense and redox regulation [12]. Glutathione peroxidases (GPx) and thioredoxin reductases are key selenoproteins that protect against oxidative stress by reducing hydrogen and lipid hydroperoxides, thus regulating cellular reactive oxygen species (ROS) levels [13].

Altered levels of zinc, copper, and selenium have been reported in the serum, synovial fluid, and cartilage of OA patients, suggesting a possible link between trace element homeostasis and disease progression [14,15]. Both deficiencies and imbalances among these elements may exacerbate oxidative damage, disturb cartilage matrix turnover, and intensify inflammatory signaling—key drivers of OA pathology. In response to the growing interest in the role of micronutrients in joint health, this literature review aims to synthesize and critically evaluate current evidence on the involvement of zinc, copper, and selenium in the pathogenesis and progression of osteoarthritis. Particular emphasis is placed on their roles in regulating oxidative stress, maintaining cartilage integrity, and modulating inflammatory responses. By integrating findings from clinical studies, experimental research, and epidemiological data, this review seeks to highlight the potential of these trace elements as biomarkers and therapeutic targets in osteoarthritis management.

## 2. Materials and Methods

A comprehensive literature search was conducted to identify relevant studies investigating the roles of zinc, copper, and selenium in osteoarthritis. The electronic databases PubMed, Scopus, and Web of Science were searched up to 4/30/2025 using combinations of the following keywords: “osteoarthritis”, “zinc”, “copper”, “selenium”, “trace elements”, “oxidative stress”, “cartilage degradation”, and “inflammation”. Studies were included if they provided clinical, experimental, or epidemiological data on the biological functions or therapeutic implications of these trace elements in the context of osteoarthritis. Articles not published in English or lacking direct relevance to the topic were excluded. The selection focused on peer-reviewed journal articles, including original research, reviews, and meta-analyses.

## 3. The Role of Essential Trace Elements in Osteoarthritis: Biological Mechanisms and Clinical Evidence

Essential trace elements are vital micronutrients that, despite being required in minute quantities, play significant roles in maintaining joint health. Their involvement in enzymatic reactions, antioxidant defense systems, immune modulation, and tissue remodeling underscores their importance in maintaining joint health and preventing degenerative conditions such as OA [7]. Among the numerous trace elements, zinc, copper, and selenium have gained particular attention due to their roles in oxidative balance, connective tissue metabolism, and inflammatory regulation. Deficiencies or imbalances of these elements have been implicated in the pathophysiology of several chronic conditions, including OA.

### 3.1. Zinc (Zn): Cartilage Regeneration and Inflammatory Modulation

Zinc is an essential trace element that plays an indispensable role in a variety of physiological and biochemical processes in the human body. It serves as a structural, catalytic, and regulatory component in approximately 300 enzymes, spanning all six enzyme classes, including zinc-dependent metalloproteinases, oxidoreductases, and dehydrogenases [16,17]. Moreover, zinc is essential for cellular metabolism, gene expression, signal transduction, immune response, and apoptosis [18,19]. As a cofactor in over 300 enzymes, it contributes to critical cellular functions such as DNA synthesis, protein production, and cell division [20]. These widespread roles highlight the systemic significance of zinc, particularly in tissues with high cellular turnover and metabolic activity.

Zinc plays a crucial role in maintaining cartilage homeostasis and regulating immune function, which has direct implications for OA pathogenesis. Zinc ions regulate intracellular signaling pathways in immune cells, with homeostasis maintained by zinc transporters and zinc-binding proteins [21]. Zinc’s anti-inflammatory and antioxidant properties are well-documented. Zinc deficiency has been associated with the development of a pro-inflammatory phenotype, which may contribute to cartilage destruction and chondrocyte apoptosis—hallmarks of OA [22]. The balance between anabolic and catabolic signaling pathways is critical for maintaining cartilage homeostasis, and disturbances in this balance contribute to joint diseases like OA [23].

One of zinc’s critical roles in joint health is its function in cellular redox homeostasis. As a cofactor in copper/zinc superoxide dismutase (Cu/Zn-SOD), zinc helps catalyze the dismutation of superoxide radicals into molecular oxygen and hydrogen peroxide, mitigating oxidative damage [24,25]. This antioxidant activity protects chondrocytes from oxidative stress-induced damage, a key driver of cartilage degradation in OA [26,27]. Moreover, in pathological states such as type 2 diabetes, altered zinc homeostasis and reduced Cu/Zn-SOD activity further link zinc deficiency to joint damage [28]. Zinc also inhibits NF-κB, a transcription factor involved in pro-inflammatory cytokine production, including TNF-α and IL-1β, thereby counteracting oxidative and inflammatory stressors contributing to OA development [18,29].

#### Clinical and Experimental Evidence

*Zinc Supplementation and Chondrocyte Function*: Zinc supplementation has demonstrated protective effects by modulating oxidative stress and chondrocyte function. In in vitro and in vivo models, zinc counteracted the damaging effects of monosodium iodoacetate (MIA) via the p-Akt/Nrf2 pathway, enhancing the expression of Nrf2 and phosphorylated Akt [30,31]. A moderate zinc dose (1.6 mg/kg/day) effectively prevented OA progression, while higher doses showed no additional benefit [30]. Additionally, zinc deficiency is associated with impaired cartilage remodeling and increased cellular senescence, further contributing to OA pathophysiology [30].

*Zinc-Based Nanotherapies*: Recent advances in zinc-based therapies also show promise. Zinc(II) enhances drug delivery systems, as seen in a Zn-driven nano-assembly delivering metformin and p65 siRNA into cartilage. Zinc’s positive charge improves cartilage retention and penetration. It promotes autophagy via the AMPK/mTOR pathway and suppresses NF-κB signaling, protecting chondrocytes from apoptosis while supporting extracellular matrix (ECM) repair through upregulation of Col2a1 and Acan [32]. This nanotherapy significantly reduced IL-6, TNF-α, and MMP-13; preserved cartilage; and improved chondrocyte viability in OA models.

*Zinc Transporters and Catabolic Pathways*: However, excessive intracellular zinc can have deleterious effects. In OA cartilage, the zinc importer ZIP8 is upregulated, resulting in increased intracellular zinc levels. This activates metal-responsive transcription factor 1 (MTF1), which binds to zinc-response elements in the promoters of catabolic genes such as MMP13 and ADAMTS5. These enzymes are central to extracellular matrix degradation. A positive feedback loop between MTF1 and the pro-degenerative factor HIF-2α further amplifies this catabolic signaling cascade, accelerating cartilage breakdown. Experimental deletion of ZIP8 or MTF1 was shown to reduce OA severity in preclinical models, highlighting this zinc-mediated pathway as a potential therapeutic target [33].

*Population and Genomic Evidence*: Epidemiological and genomic studies support zinc’s involvement in OA risk. A large NHANES-based cross-sectional study found that higher daily zinc intake, along with copper and selenium, was significantly associated with increased OA risk, suggesting that excessive intake may be detrimental [15]. This may reflect a U-shaped relationship, where both deficiency and excess are detrimental. Additionally, unmeasured confounding factors, such as supplement overuse, comorbid conditions, or dietary interactions, may partially explain this association. Consistently, Mendelian randomization using GWAS data revealed a strong causal link between elevated serum zinc levels and increased risk of knee and spine OA, likely through promotion of matrix-degrading enzyme activity [34,35]. These findings position zinc as a potential biomarker and therapeutic target in OA.

*Tissue Zinc Levels and Epigenetic Changes*: Conversely, some studies report lower zinc levels in OA patients. Reduced zinc is linked with impaired cartilage integrity and heightened degeneration, emphasizing the importance of maintaining optimal zinc levels for joint health [30,36]. In hip OA, patients showed increased zinc content in femoral bone with disease severity, implicating zinc in OA progression and matrix degradation through its role as an MMP cofactor [37]. A subgroup of hip OA patients exhibited epigenetic and transcriptomic changes, including upregulation of MTF1 and its targets MMP13 and ADAMTS5, supporting the zinc–inflammation–matrix degradation connection [38].

*Zinc Combinations and Biomaterials*: In therapeutic development, zinc has shown efficacy in combination treatments. Zinc combined with a probiotic complex and rosavin reduced OA progression in MIA-induced rat models by suppressing pro-inflammatory cytokines and catabolic gene expression in patient-derived chondrocytes [39]. Zinc folate (ZnFO)-loaded scaffolds promoted cartilage matrix metabolism and gene expression (e.g., COL2A1, SOX9), with sustained zinc release supporting cell proliferation and differentiation. These scaffolds facilitated cartilage and subchondral bone repair in vivo [40]. Similarly, ZnO nanoparticle-based biodegradable scaffolds enhanced osteochondral differentiation of mesenchymal stem cells (MSCs), with dose-dependent promotion of chondrogenic and osteogenic markers [41].

*Zinc-Related Genetic Markers*: Furthermore, zinc metabolism-related genes (ZMRGs) have been identified in OA. Genes such as MMP2, MMP3, MMP9, and MMP13 were upregulated and associated with disease progression, making them potential biomarkers and therapeutic targets [42].

**Figure 1:**
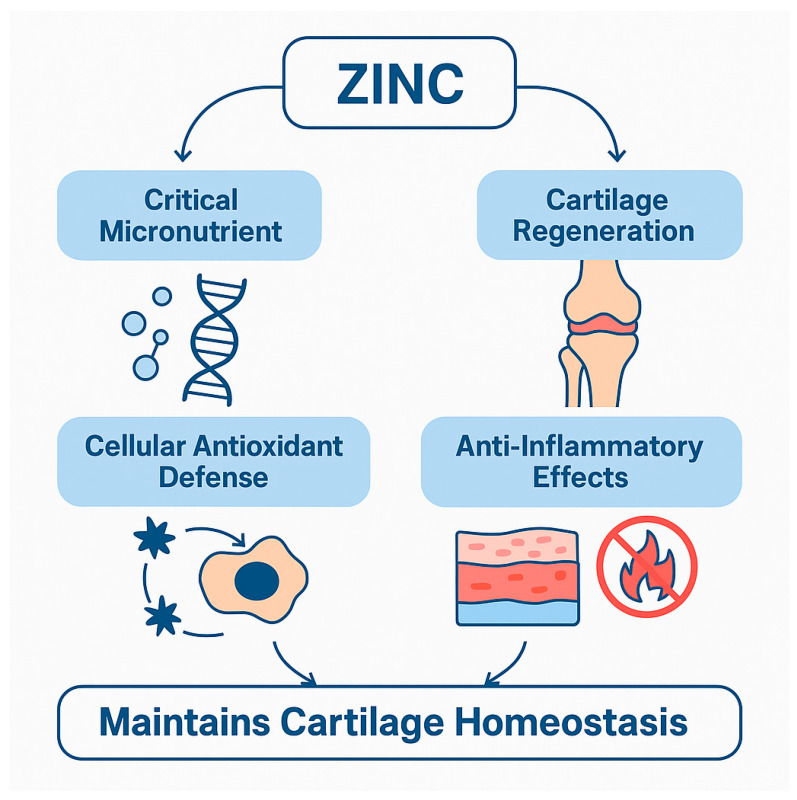
Zinc mediates cartilage regeneration and inflammation control through its roles as a critical micronutrient, antioxidant, and anti-inflammatory agent. These mechanisms converge to maintain cartilage homeostasis and modulate OA progression.

The multifaceted role of zinc in osteoarthritis is visually summarized in Figure 1, which illustrates its contributions to antioxidant defense, inflammation modulation, and cartilage repair. Additionally, Table 1 consolidates experimental and clinical findings across multiple domains, highlighting key molecular targets and pathways influenced by zinc. Together, these tools provide a comprehensive overview of how zinc homeostasis impacts OA progression and treatment strategies.

### 3.2. Copper (Cu): Structural, Antioxidant, and Immunomodulatory Roles in OA

Copper is an indispensable trace element that plays a pivotal role in maintaining the structural integrity of connective tissues, particularly cartilage and bone. One of its primary functions is serving as a cofactor for lysyl oxidase (LOX), an enzyme crucial for the cross-linking of collagen and elastin fibers in the ECM. This cross-linking process is essential for the tensile strength and elasticity of cartilage, attributes that are compromised in osteoarthritic conditions. Studies have demonstrated that dietary copper levels directly influence LOX activity, with deficiencies leading to impaired collagen cross-linking and subsequent weakening of cartilage structure [43,44,45,46]. Furthermore, copper is involved in the post-translational modification of LOX, facilitating the formation of its active site cofactor, lysyl tyrosylquinone, which is vital for its enzymatic activity [47].

Beyond its structural roles, copper is integral to the body’s antioxidant defense mechanisms. In parallel with zinc, copper contributes to SOD-mediated oxidative defense in chondrocytes. Oxidative stress is a significant contributor to chondrocyte apoptosis and cartilage degradation in OA. Disruptions in copper homeostasis can lead to decreased Cu/Zn-SOD activity, resulting in increased ROS production and mitochondrial dysfunction. Additionally, copper’s involvement in redox balance plays a role in preventing ferroptosis, a form of iron-dependent cell death associated with lipid peroxidation in chondrocytes [48].

Copper’s role in inflammatory regulation overlaps with zinc, particularly in modulating IL-1β and TNF-α levels [49]. Conversely, copper deficiency impairs immune responses, weakens antioxidant defenses, and disrupts mitochondrial function. These effects have been demonstrated in animal models, where copper-depleted diets lead to both humoral and cell-mediated immunosuppression, and supported by human studies showing decreased ceruloplasmin activity and neutropenia in copper-deficient individuals [50,51]. Given that OA is characterized by chronic low-grade inflammation, maintaining optimal copper balance is essential not only for structural and antioxidant defense but also for controlling immune-mediated joint degradation.

#### Clinical and Experimental Evidence

*Serum and Tissue Copper Levels in OA*: Numerous studies have explored copper concentrations in biological compartments of OA patients. Elevated serum and plasma copper levels have been linked with inflammatory markers and OA risk [52,53]. Higher copper concentrations have also been observed in joint tissues such as femoral heads and menisci in advanced OA stages, indicating localized accumulation during disease progression [37,54]. Dietary studies have demonstrated a relationship between high copper intake and increased OA risk, although serum levels may not always directly reflect intake due to regulatory imbalances [15]. Meta-analytic data further reinforce the observation that circulating copper levels are consistently higher in OA patients compared to healthy controls [14]. However, some research suggests lower copper levels may be associated with OA [36,55]. This highlights the complex role of copper in bone and joint health.

The contradictory findings likely reflect variability across populations due to factors like geography, diet, and metabolism. For instance, a study found higher serum copper in picky eaters among children, demonstrating how dietary habits can influence trace element levels [56]. Additionally, the study showed that high iron intake can lead to copper deficiency, illustrating how nutrient interactions may impact copper status [57].

*Copper Supplementation and Toxicity Risk*: Copper-based nanotherapeutics offer novel approaches for OA treatment. Various nanoparticle systems, such as B2M-CuS [58], CSP@AS-IV [59], and Cu-indomethacin gels [60], have demonstrated anti-inflammatory, antioxidant, and regenerative effects in preclinical OA models. Other delivery platforms, including injectable PMs@CuBG microspheres [61], D-CuS@NR nanoparticles [62], and MSCs@CuS@CDKN1A systems [63], have shown effectiveness in modulating immune responses, promoting chondrocyte survival, and enhancing ECM synthesis. These copper-enabled technologies illustrate the potential for safe, localized, and multi-modal OA therapies.

*Immunoinflammatory Mechanisms*: The immunoregulatory functions of copper extend to modulating macrophage phenotypes and downregulating matrix-degrading enzymes. Studies have shown that copper-incorporated bioactive ceramics can shift macrophage polarization toward an anti-inflammatory profile while suppressing catabolic activity in inflamed cartilage [64].

*Genetic and Molecular Insights*: Copper’s influence in OA is also evident at the molecular level. Copper transporter gene variants (e.g., ATP7A, ATP7B) have been associated with systemic inflammation and mineral regulation, though causal mechanisms remain to be fully elucidated. Emerging evidence suggests that dysregulation of cuproptosis-related genes may contribute to OA pathogenesis, with key genes such as FDX1, DLAT, and MTF1 potentially involved [48,65]. Cuproptosis, a newly characterized form of regulated cell death, is triggered by intracellular copper accumulation and aggregation of lipoylated mitochondrial enzymes, ultimately leading to proteotoxic stress [66]. While direct evidence in OA is limited, this mechanism may represent a novel pathway affecting chondrocyte survival under copper imbalance. Furthermore, Mendelian randomization studies provide supportive genetic evidence that higher circulating copper levels may causally contribute to OA susceptibility [67,68].

**Figure 2:**
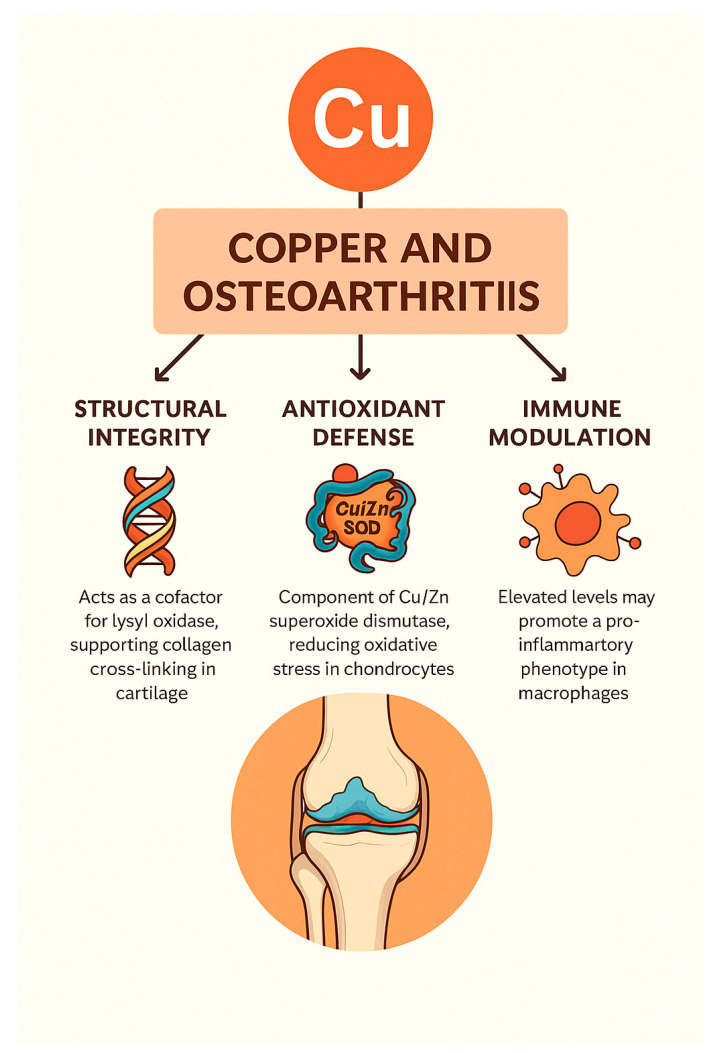
Overview of copper’s roles in osteoarthritis. This infographic illustrates the interconnected roles of copper in osteoarthritis pathogenesis and therapy. It highlights copper’s involvement in ECM stability, redox regulation, immune modulation, ferroptosis prevention, genetic control, and nanotherapeutic delivery, emphasizing its dual impact on joint degeneration and regeneration.

In general, copper exerts multifaceted effects in the pathophysiology and potential treatment of OA, influencing processes related to structural matrix stability, oxidative stress regulation, immune modulation, and gene-mediated pathways such as cuproptosis. Both copper deficiency and excess have been associated with cartilage degradation, inflammation, and chondrocyte death. Advances in copper-targeted therapeutics—including nanoparticles and bioactive delivery systems—offer promising avenues for restoring joint homeostasis and attenuating disease progression. These mechanistic domains and therapeutic implications are summarized in Table 2 and visually represented in Figure 2.

### 3.3. Selenium (Se): Redox and Immune-Modulatory Roles in Osteoarthritis

Selenium is an essential trace element that exerts its biological effects primarily through its incorporation into a family of selenoproteins, including GPx and thioredoxin reductases (TrxR), which play indispensable roles in redox homeostasis. Selenium supports redox homeostasis through selenoproteins, complementing the antioxidant effects of zinc and copper [69,70,71,72]. The antioxidant function of selenium is particularly significant in OA joints, where oxidative stress is a known contributor to cartilage degradation and disease progression [73].

Selenium, like zinc and copper, influences NF-κB signaling in OA joints through its role in regulating oxidative stress [74,75]. By mitigating these inflammatory pathways, selenium contributes to a more balanced immune microenvironment within the joint, potentially slowing OA progression. Moreover, selenium is involved in regulating immune cell function, including neutrophil activity and T-cell proliferation, thereby reducing the risk of chronic inflammation and joint tissue damage [76].

Several clinical and experimental studies have demonstrated that selenium deficiency is associated with increased susceptibility to inflammatory joint diseases, including Kashin–Beck disease and OA [77,78]. Low selenium levels in synovial fluid and serum have been correlated with increased oxidative stress markers, elevated inflammatory cytokines, and greater cartilage damage in OA patients [79]. These findings underscore the therapeutic potential of selenium supplementation or selenoprotein modulation in preserving cartilage integrity and controlling inflammation in OA.

#### Mechanistic, Therapeutic, and Population-Level Evidence

*Antioxidant Signaling and Genetic Regulation*: Reduced levels of selenoproteins such as selenoprotein P (SELENOP) and glutathione peroxidase 3 (GPx3) have been identified in OA patients despite comparable serum selenium concentrations. These deficiencies correlate with poorer functional performance and may reflect inflammation-induced suppression of hepatic SELENOP and impaired renal GPx3 synthesis [80]. Moreover, polymorphisms in selenium-responsive genes, such as GPX1, SELENOS, DIO2, PPARG, SMAD3, ADAM12, and TIMP2, have been linked to increased susceptibility to OA and Kashin–Beck disease by disrupting redox signaling and extracellular matrix (ECM) homeostasis [81,82]. These molecular alterations highlight selenium’s role in modulating oxidative defense, transcriptional regulation, and genetic risk.

*Chondrogenesis, DNA Repair, and Cartilage Anabolism*: Selenium deficiency impairs chondrogenesis by downregulating anabolic markers such as SOX9, COL2A1, and aggrecan. In contrast, selenium supplementation restores these gene expressions and promotes cartilage formation and matrix stability. It also enhances DNA repair capacity and shields chondrocytes from oxidative genomic damage, thereby preserving their proliferative and regenerative functions in degenerative joint environments [82].

*Cellular Stress Response and Matrix Preservation*: Selenium may help maintain chondrocyte viability by supporting mitochondrial integrity, reducing ROS accumulation, and modulating redox-sensitive signaling pathways (e.g., Nrf2, PI3K/Akt, JNK, Wnt/β-catenin). It also suppresses catabolic and inflammatory mediators such as MMP13, TNF-α, IL-1β, COX-2, and iNOS while supporting type II collagen synthesis [7,8,73,77,82,83]. Notably, selenium has been shown to influence glycosylation patterns in chondrocytes derived from patients with Kashin–Beck disease, suggesting a potential role in the post-translational regulation of matrix-associated proteins and cartilage homeostasis [83]. These mechanisms collectively contribute to matrix preservation and suppression of chondrocyte apoptosis.

*Innovative Nanomedicine Strategies for OA*: Recent advances in nanotechnology have enabled the development of selenium-based delivery platforms with enhanced therapeutic efficacy. Selenium nanoparticles (SeNPs) have demonstrated significant anti-inflammatory and chondroprotective effects in in vitro and animal models, including downregulation of IL-1β–induced pro-inflammatory genes and catabolic enzymes (e.g., MMP13, ADAMTS-5), upregulation of ECM components (COL2A1, aggrecan), and modulation of NF-κB and MAPK signaling pathways [75].

**Figure 3:**
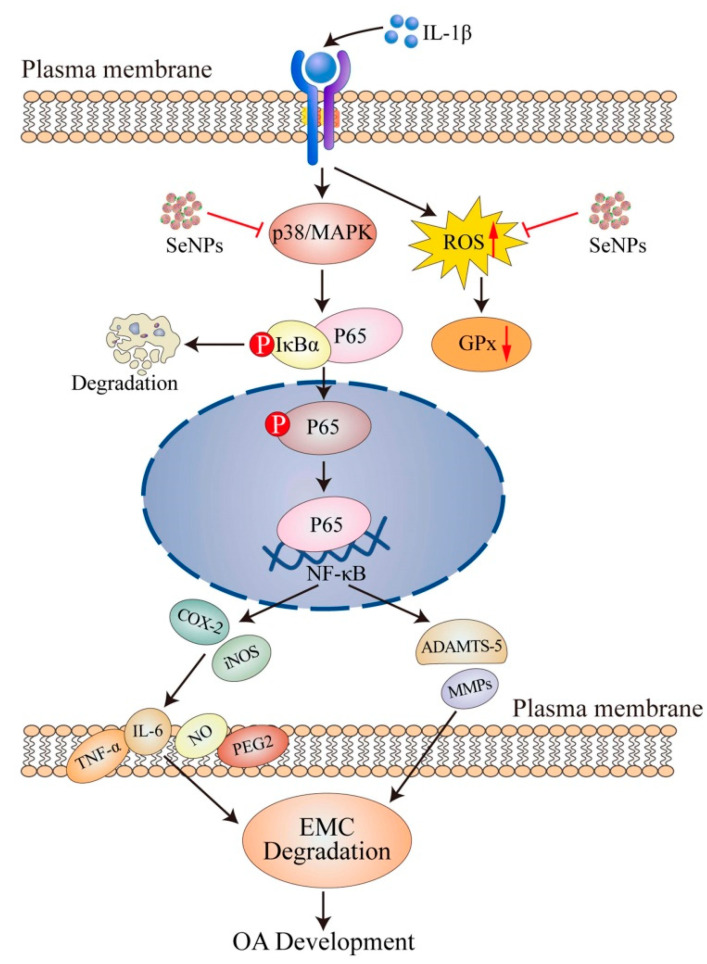
Protective effects of selenium nanoparticles (SeNPs) in osteoarthritis. SeNPs suppress IL-1β-induced acti-vation of the p38/MAPK and NF-κB signaling pathways in chondrocytes. Notably, IL-1β stimulation increases reac-tive oxygen species (ROS↑) and decreases glutathione peroxidase activity (GPx↓), contributing to oxidative stress and inflammation. SeNPs mitigate these effects by reducing ROS levels and restoring GPx activity. This modulation leads to the downregulation of iNOS, COX-2, IL-6, and catabolic enzymes (MMP-13, ADAMTS-5), while promoting the expression of COL2A1 (type II collagen) and aggrecan, thereby protecting against extracellular matrix (ECM) degradation and osteoarthritis (OA) progression. Abbreviations: OA, osteoarthritis; SeNPs, selenium nanoparticles; IL, interleukin; MAPK, mitogen-activated pro-tein kinase; ROS, reactive oxygen species; GPx, glutathione peroxidase; IκB, inhibitor of kappa B; NF-κB, nuclear factor κB; ACAN, aggrecan; COL-2, type II collagen; ADAMTS, a disintegrin and metalloproteinase thrombospon-din motifs; MMP, matrix metalloproteinase; NO, nitric oxide; iNOS, inducible nitric oxide synthase; COX-2, cy-clooxygenase-2 [75].

Coating SeNPs with polydopamine (PDA-SeNPs) further improved their biocompatibility, antioxidant potential, and chondrogenic activity, while suppressing oxidative stress and canonical Wnt signaling inhibition [84]. Dual-scale targeted systems—such as HA-SeNPs@AHAMA-HMs—have demonstrated controlled release, enhanced cartilage affinity, and robust selenoprotein reactivation in preclinical OA models, contributing to structural preservation of cartilage [85]. While these nanotherapeutics represent a promising research direction, their clinical efficacy and safety remain to be established through human trials. The therapeutic framework and biological outcomes of SeNP administration in OA are visually summarized in Figure 3.

*Epidemiologic Correlates and Risk Modification*: Multiple studies have confirmed the association between selenium status and OA risk. Mendelian randomization studies suggest a potential causal inverse relationship between serum selenium levels and OA incidence, particularly among women [86]. Cross-sectional analyses from Nigeria and other regions further report significantly lower selenium levels in OA patients, suggesting selenium deficiency contributes to disease vulnerability and may warrant targeted nutritional or therapeutic intervention [15,36]. These findings emphasize selenium’s value not only as a therapeutic agent but also as a biomarker for OA risk stratification.

Collectively, selenium exerts multifaceted effects on OA through a range of biological mechanisms. It modulates antioxidant signaling by regulating key selenoproteins such as GPX1 and SELENOP and influences redox-sensitive pathways including NF-κB, PI3K/Akt, and TGF-β. Genetic variations in selenium-regulating genes (e.g., GPX1, SELENOS, PPARG) further implicate selenium in cartilage homeostasis and oxidative defense. Selenium also promotes chondrogenesis and DNA repair by enhancing SOX9, COL2A1, and aggrecan expression while protecting against genomic instability. At the cellular level, selenium restores mitochondrial function, reduces oxidative stress and inflammation, and supports matrix preservation. Advances in nanomedicine have leveraged selenium’s bioactivity to develop SeNP-based delivery systems that target inflamed joints and promote cartilage repair. Finally, population-based studies and Mendelian randomization analyses support a protective role for selenium against OA risk, particularly among selenium-deficient individuals. These domains are comprehensively summarized in Table 3.

## 4. Combined Effects and Interactions

While zinc, copper, and selenium have individually demonstrated significant roles in OA pathogenesis and progression, emerging evidence highlights the importance of examining their interrelated effects. These trace elements are intricately connected through shared involvement in key physiological processes such as redox homeostasis, inflammatory signaling, and ECM remodeling. For instance, zinc and copper are both critical cofactors for the antioxidant enzyme Cu/Zn-SOD, and their ratio is known to influence enzyme stability and activity [25,26,48]. Similarly, selenium is required for the synthesis of GPxs, which function alongside Cu/Zn-SOD in mitigating oxidative stress, particularly in chondrocytes [70,72].

Nutrient–nutrient interactions play a critical role in maintaining trace element homeostasis. Zinc and copper exhibit a complex interplay in trace element homeostasis, with significant implications for various physiological processes, including those relevant to OA. The divalent metal transporter-1 (DMT1) plays a crucial role in the absorption of both zinc and copper, as well as other metals like iron [87,88]. This shared transport mechanism creates potential for competitive interactions between these metals. Excessive zinc intake can induce the expression of metallothionein, a protein that preferentially binds copper, thereby reducing its systemic absorption [89,90]. This antagonistic relationship between zinc and copper can lead to imbalances in their homeostasis, potentially impacting various bodily functions.

The balance among these trace elements appears to be more important than their absolute levels. Differential concentrations of zinc and copper in joint tissues have been observed, suggesting a potential antagonistic relationship [54]. Elevated zinc-to-copper ratios have also been linked to the radiographic severity of OA, emphasizing the pathological relevance of their relative proportions [37]. Additionally, selenium’s ability to modulate both NF-κB and Nrf2 signaling pathways enables it to indirectly influence the expression and activity of enzymes regulated by copper and zinc. For example, the Nrf2–antioxidant response element (ARE) pathway governs transcription of selenoproteins (e.g., GPX1), Cu/Zn-SOD, and metallothioneins, while NF-κB coordinates redox-sensitive inflammatory gene expression, highlighting these shared regulatory networks [73].

Despite these insights, the mechanistic interplay among these elements remains underexplored in human populations. Most existing studies focus on monotherapies, and few clinical trials have examined multi-element supplementation with controlled dosage and duration. Additionally, factors such as age, comorbidities, dietary patterns, and genetic polymorphisms can influence the bioavailability and metabolism of these trace elements, further complicating their interaction dynamics. Overall, these trace elements likely operate as part of a dynamic micronutrient network that modulates inflammation, oxidative damage, and cartilage metabolism in OA. Future interventions may benefit from a systems biology approach, integrating micronutrient profiling, gene–nutrient interactions, and clinical phenotyping to personalize trace element–based therapies for OA.

## 5. Challenges and Controversies

While clinical and experimental evidence supports the involvement of zinc, copper, and selenium in OA, several challenges complicate interpretation. Heterogeneity in study design, patient demographics, and measurement techniques often leads to conflicting findings. For example, discrepancies between serum, plasma, and synovial fluid concentrations of trace elements can affect outcome interpretation [15,36,54,73].

Additionally, biological variables such as age, sex, dietary habits, and comorbidities may influence trace element homeostasis, reducing the generalizability of study results [37,52]. The lack of large-scale, randomized controlled trials (RCTs) with long-term follow-up further limits the ability to determine causal relationships and therapeutic efficacy. Several trials assessing the clinical efficacy of trace element supplementation have reported null results or limited benefits, highlighting the need for cautious interpretation of supplementation effects.

Moreover, comorbid conditions such as diabetes mellitus, obesity, and metabolic syndrome can alter trace element metabolism and may confound associations with OA outcomes [91]. For example, selenium status has been linked to insulin resistance and components of metabolic syndrome, including hyperglycemia and hypertension, in a large epidemiological study [92]. Similarly, zinc deficiency is more prevalent in individuals with hyperglycemia or prediabetes, and altered zinc status correlates with impaired insulin secretion and resistance [93]. These interactions underscore the importance of evaluating trace element effects within the broader context of metabolic health.

These limitations underscore the inherent complexity of micronutrient research in OA and highlight the need for standardized assessment methods and cautious interpretation of current data.

### 5.1. Conflicting Clinical Evidence

While growing evidence supports a role for zinc, copper, and selenium in osteoarthritis, the literature also presents considerable inconsistencies. Conflicting findings, such as variations in trace element concentrations between serum, plasma, and synovial fluid, complicate interpretation and highlight methodological limitations. Factors including geographic variation, dietary habits, sample collection methods, and analytical techniques contribute to this heterogeneity. These issues underscore the need for critical evaluation of existing data and the implementation of standardized research protocols.

The clinical evidence concerning trace elements such as zinc, copper, and selenium in OA remains inconsistent, presenting a challenge for the identification of reliable biomarkers. Some studies indicate reduced serum zinc levels in OA patients, suggesting a potential deficiency or altered metabolism in these individuals [15]. Conversely, other research reports elevated zinc concentrations in synovial fluid, which may be linked to the activation of matrix-degrading enzymes contributing to cartilage destruction in OA [94,95]. Similar inconsistencies are evident for copper and selenium. Elevated copper levels have been observed in the synovial fluid of OA patients and may reflect underlying inflammatory processes [96,97]. Selenium levels also show mixed patterns; while some studies report no significant differences in serum selenium levels between OA and non-arthritis groups, others note decreased levels in inflammatory conditions [15,96].

While these inconsistencies complicate the development of diagnostic tools, they also highlight the metabolic complexity of trace elements in OA. Broader, standardized, and methodologically rigorous studies are needed to clarify their role in OA pathogenesis and to support the development of reliable biomarkers for diagnosis and prognosis.

### 5.2. Bioavailability and Absorption Factors

The bioavailability and absorption of trace elements such as zinc, copper, and selenium are regulated by multiple physiological, dietary, and pathological factors, all of which are particularly relevant when considering supplementation strategies for older adults with OA. One key mechanism is competitive inhibition at shared transporters. For instance, zinc and copper utilize overlapping transport pathways, and high zinc intake can inhibit copper absorption by out-competing it at intestinal transport sites [98,99]. The copper transporter CRT1 is notably upregulated in states of dietary copper deficiency, further reflecting the dynamic regulation of these elements based on nutritional status [99].

Additionally, the chemical form of a trace element significantly influences its absorption. Organic forms of selenium, such as selenomethionine and selenocysteine, are more efficiently absorbed than inorganic forms like selenate, largely due to their transport via amino acid carriers [98,100]. However, unlike other essential nutrients, these organic selenium compounds lack tight homeostatic control mechanisms, making their supplementation potentially more variable in effect [99]. Age-related changes in gastrointestinal physiology further complicate absorption. Older adults may experience reduced gastric acid production and altered intestinal permeability, which can negatively impact the uptake of zinc, copper, and selenium [98].

### 5.3. Safety and Toxicity Concerns

While supplementation can be an effective strategy to correct deficiencies in zinc, copper, and selenium, it must be approached cautiously due to the narrow therapeutic windows associated with these elements. Excessive intake, particularly when administered without consideration of individual absorption capacity and metabolic state, can result in toxicity. For example, chronic overexposure to selenium or copper has been associated with oxidative stress, liver damage, and metabolic disturbances, emphasizing the importance of personalized dosing regimens [101].

Given the interdependent absorption pathways and variable bioavailability influenced by age, disease, and chemical form, supplementation must be tailored to individual needs. This requires regular monitoring of trace element status and careful selection of dosage and formulation. Particularly in older adults or those with comorbid conditions such as OA, a nuanced understanding of trace element metabolism is essential to avoid adverse effects while maximizing therapeutic benefits.

## 6. Future Directions

Despite growing interest in the role of trace elements in OA, critical knowledge gaps remain that hinder the development of evidence-based clinical applications. Bridging these gaps will require interdisciplinary collaboration, standardized methodologies, and a concerted effort to move beyond descriptive studies toward mechanistic and translational research.

*Large-Scale Randomized Controlled Trials (RCTs)*: To establish causal relationships and determine the clinical efficacy of trace element supplementation, robust randomized controlled trials are urgently needed. The current evidence base is constrained by small sample sizes, heterogeneous study designs, short intervention durations, and non-standardized outcome measures. Well-powered, methodologically rigorous RCTs are essential to define optimal dosages, treatment durations, and responsive patient subgroups. Furthermore, trials should integrate mechanistic endpoints to elucidate the biological pathways through which trace elements exert their effects in OA.

Despite compelling preclinical data, the translation of zinc, copper, and selenium research into clinical applications for OA remains limited. Few RCTs have evaluated the efficacy of supplementation, and existing studies often lack standardized endpoints or consistent dosages. The absence of validated biomarkers for trace element status in joint tissues hampers both diagnosis and treatment monitoring. Currently, there is also insufficient dose–response data, making it difficult to determine optimal intake levels or therapeutic thresholds for these elements.

*Personalized Nutrition and Nutrigenomics*: Advances in nutrigenomics and precision medicine offer new opportunities to tailor trace element interventions based on genetic predispositions, metabolic phenotypes, and baseline nutritional status. Interindividual variability in absorption, distribution, and metabolism of zinc, copper, and selenium is likely to influence both disease susceptibility and response to supplementation, though further studies are needed. Integrating genomic, biochemical, and lifestyle data could optimize supplementation strategies, minimize adverse effects, and enhance patient outcomes through a more individualized approach.

*Development of Standardized Biomarkers*: A major barrier to progress is the absence of reliable, validated biomarkers for trace element status in OA. Future research should prioritize the identification and validation of sensitive and specific biomarkers—whether in serum, synovial fluid, or cartilage tissue—that can be used for early diagnosis, disease monitoring, and treatment stratification. Biomarker standardization will also be critical for cross-study comparisons and the advancement of personalized interventions.

*Investigating Synergistic and Multimodal Therapies*: Given the multifactorial nature of OA, future studies should explore the potential of multimodal interventions that combine trace element supplementation with other bioactive compounds, such as vitamin D, omega-3 fatty acids, polyphenols, or glucosamine. Investigating synergistic effects at molecular and clinical levels may yield more comprehensive and durable benefits, potentially modifying disease progression rather than solely alleviating symptoms.

*Emerging Technologies and Monitoring Tools*: In addition, recent advances in digital health and nanotechnology offer promising tools for the real-time monitoring and delivery of trace elements. Artificial intelligence (AI)-based nutrient tracking platforms and lab-on-chip biosensors may provide individualized assessments of micronutrient status, supporting timely and targeted interventions in OA management. These technologies can enhance diagnostic precision, improve compliance, and enable continuous patient monitoring in both clinical and community settings.

## 7. Conclusions

Zinc, copper, and selenium are essential trace elements with integral roles in biological processes implicated in OA pathogenesis, including oxidative stress, inflammation, and extracellular matrix degradation. An expanding body of preclinical and observational evidence suggests that imbalances in these micronutrients may influence the onset and progression of OA. However, the clinical translation of these findings remains limited by methodological inconsistencies, inadequate sample sizes, and a lack of mechanistic insight.

Incorporating trace element assessment and targeted supplementation into OA management holds promise as a low-cost, adjunctive therapeutic strategy. However, achieving clinical efficacy will require a shift toward precision-based approaches, supported by robust biomarker development and individualized treatment protocols. Future research must strive to delineate the mechanistic underpinnings of trace element function in joint biology and to evaluate the long-term safety and effectiveness of supplementation strategies through well-designed clinical trials. Advancing our understanding of elemental homeostasis in OA may ultimately facilitate the development of personalized interventions that enhance disease management and improve quality of life. These insights may also guide the next generation of mechanism-based strategies for osteoarthritis prevention and treatment.

## Figures and Tables

**Table 1:** Key roles of zinc in osteoarthritis based on functional domains and experimental evidence.

Functional Role	Mechanism/Target	Outcome in OA	Reference(s)
Antioxidant Defense	Cofactor in Cu/Zn-SOD	Reduces oxidative stress and cell damage	[24,25,26,27]
Anti-Inflammatory Modulation	Inhibits the NF-κB pathway and reduces cytokines (TNF-α, IL-1β)	Decreases inflammation, slows progression	[18,29]
Chondrocyte Protection	Activates the p-Akt/Nrf2 pathway	Enhances viability, reduces apoptosis	[30,31]
Matrix Degradation Control	Modulates MMP13, ADAMTS5 via ZIP8-MTF1 axis	Slows ECM breakdown	[33,37,38]
Genetic/Transcriptomic Regulation	Upregulation of MMPs (MMP2, MMP3, MMP9, MMP13)	Associated with OA risk and progression	[42]
Regenerative Therapy	Zinc-loaded scaffolds (ZnFO, ZnO NPs)	Promotes cartilage/bone regeneration	[40,41]
Dietary Influence	Excess zinc intake, high serum Zn levels	Linked to increased OA risk	[15,34]
Combination Therapy	Zinc + probiotics/rosavin	Reduces cytokines, protects cartilage	[39]
Bone Zinc Accumulation	Increased Zn in femoral bone	Correlates with disease severity	[37]

**Table 2:** Key roles of copper in osteoarthritis based on functional domains and experimental evidence.

Functional Domain	Role of Copper	OA-Relevant Outcomes	Reference(s)
ECM Integrity	Cofactor for lysyl oxidase (LOX)	Maintains collagen cross-linking and cartilage structure	[43,44,45,46,47]
Antioxidant Defense	Component of Cu/Zn-SOD	Reduces ROS and protects chondrocytes from oxidative stress	[48]
Ferroptosis Regulation	Regulates redox homeostasis and prevents lipid peroxidation	Prevents chondrocyte death and degeneration	[48]
Inflammation Modulation	Influences cytokine production, macrophage polarization	Controls joint inflammation and immune balance	[49,50,64]
Serum and Tissue Levels	Elevated or deficient levels linked with OA severity	Indicates copper’s role in OA risk and progression	[14,15,36,37,52,53,54]
Nanotherapeutic Delivery	B2M-CuS, CSP@AS-IV, Cu-Indo gel, PMs@CuBG, etc.	Enhances targeted therapy, ECM synthesis, and cartilage repair	[58,59,60,61,62,63]
Genetic Associations	Transporter gene variants, cuproptosis-related genes	Contribute to susceptibility and disease mechanisms	[48,65,67,68]

**Table 3:** Summary of selenium’s multifaceted roles in osteoarthritis pathogenesis and therapy. This table outlines the major biological domains through which selenium exerts its influence on OA, highlighting its molecular functions, therapeutic mechanisms, tissue-level distribution, and population-level associations. Each domain is supported by evidence from in vitro, in vivo, and clinical studies.

Domain	Key Functions	References
Antioxidant Signaling and Genetic Regulation	Modulates selenoproteins (GPX1, SELENOP); regulates NF-κB, PI3K/Akt, and TGF-β pathways; SNPs in GPX1, SELENOS, DIO2, PPARG, SMAD3, and ADAM12 influence redox and ECM homeostasis.	[80,81,82]
Chondrogenesis and DNA Repair	Enhances SOX9, COL2A1, and aggrecan expression; supports cartilage development and DNA repair mechanisms; protects against oxidative genomic instability.	[7,82]
Cellular Stress Response and Matrix Preservation	Restores mitochondrial function; reduces ROS, MMP13, IL-1β, and COX-2; supports glycosylation changes; attenuates oxidative stress and apoptosis.	[73,77,83]
Innovative Nanomedicine Strategies	SeNPs, PDA-SeNPs, and HA-SeNPs modulate inflammation and ECM degradation; enhance antioxidant activity and cartilage repair; regulate NF-κB, MAPK, and Wnt pathways; and enable targeted delivery.	[8,75,84,85]
Epidemiologic Risk Modification	Inverse association of Se status with OA risk; genetic MR studies; low Se in OA populations; sex-specific protective effects.	[15,36,86]
